# The optimal duration of anti-tuberculous therapy before pericardiectomy in constrictive tuberculous pericarditis

**DOI:** 10.1186/s13019-021-01691-9

**Published:** 2021-10-26

**Authors:** Likui Fang, Guocan Yu, Bo Ye, Fangming Zhong, Gang Chen

**Affiliations:** Department of Thoracic Surgery, Hangzhou Red Cross Hospital, Hangzhou, 310003 China

**Keywords:** Anti-tuberculous therapy, Pericardiectomy, Constrictive tuberculous pericarditis

## Abstract

**Background:**

It is unclear about the duration of anti-tuberculous therapy before pericardiectomy (DATT) in the patients with constrictive tuberculous pericarditis. This study aims to explore the optimal DATT and its impact on surgical outcomes in these patients.

**Methods:**

We retrospectively enrolled 93 patients with constrictive tuberculous pericarditis undergoing pericardiectomy and divided them into two groups according to the optimal cutoff value of DATT which was determined by the receiver operating characteristic (ROC) curve and Youden Index. Postoperative and survival outcomes were compared between the two groups.

**Results:**

The optimal cutoff value of DATT was 1.05 (months). The enrolled patients were divided into the DATT ≤ 1.05 group and the DATT > 1.05 group, with 24 (25.8%) and 69 (74.2%) cases, respectively. Comparing with the DATT ≤ 1.05 group, the DATT > 1.05 group had shorter postoperative ICU stay (*P* = 0.023), duration of chest drainage (*P* = 0.002), postoperative hospital stay (*P* = 0.001) and lower incidence of postoperative complications (*P* < 0.001). There were no statistical differences between the two groups in recurrence and survival outcomes.

**Conclusions:**

It would be of potential benefit to enhance recovery after pericardiectomy if DATT lasted for at least 1 month in the patients with constrictive tuberculous pericarditis.

## Introduction

Although positive public health approaches are implemented in most countries, tuberculosis remains one of the most common infectious diseases and a major cause of death in the world [1, 2]. In developing countries, tuberculosis is the dominant cause of pericarditis [3–5]. Due to the chronic inflammation of tuberculous pericarditis, pericardium becomes fibrotic, thickened and inelastic which inhibits the ventricular filling and eventually leads to constrictive pericarditis even after standard anti-tuberculous therapy (ATT) [6, 7].

Constrictive tuberculosis pericarditis is associated with poor treatment outcomes of medication therapy [8, 9]. Surgical intervention is recommended if there is persistent pericardial constriction and pericardiectomy is the standard surgical method to relieve the constriction [9–11]. Although pericardiectomy is regarded as the most effective treatment of constrictive tuberculous pericarditis, it is accompanied with high risk of postoperative complications and mortality [12]. There have been some studies reporting the possible risk factors of poor outcomes after pericardiectomy, but no one focuses on the duration of anti-tuberculous therapy before pericardiectomy (DATT). This study aims to explore the optimal DATT and its impacts on surgical outcomes in the patients with constrictive tuberculous pericarditis.

## Materials and methods

### Patients selection

The records of all patients with constrictive tuberculous pericarditis in our department were retrospectively reviewed from November 2012 to November 2019. The patients who underwent pericardiectomy were included in this study. Finally, a total of 93 patients were enrolled. Their clinical characteristics including DATT were collected from the hospital electronic medical records system. The study protocol was approved by the Institutional Review Board of Hangzhou Red Cross Hospital.

### Treatment

All patients were recommended to receive the standard first-line anti-tuberculous regimen (isoniazid, rifampin, pyrazinamide, and ethambutol) [13], but if patients could not tolerate these drugs, the regimen was adjusted according to individual conditions. Percutaneous internal jugular vein puncture and catheterization were routinely performed preoperatively to monitoring the central venous pressure (CVP).

**Fig. 1:**
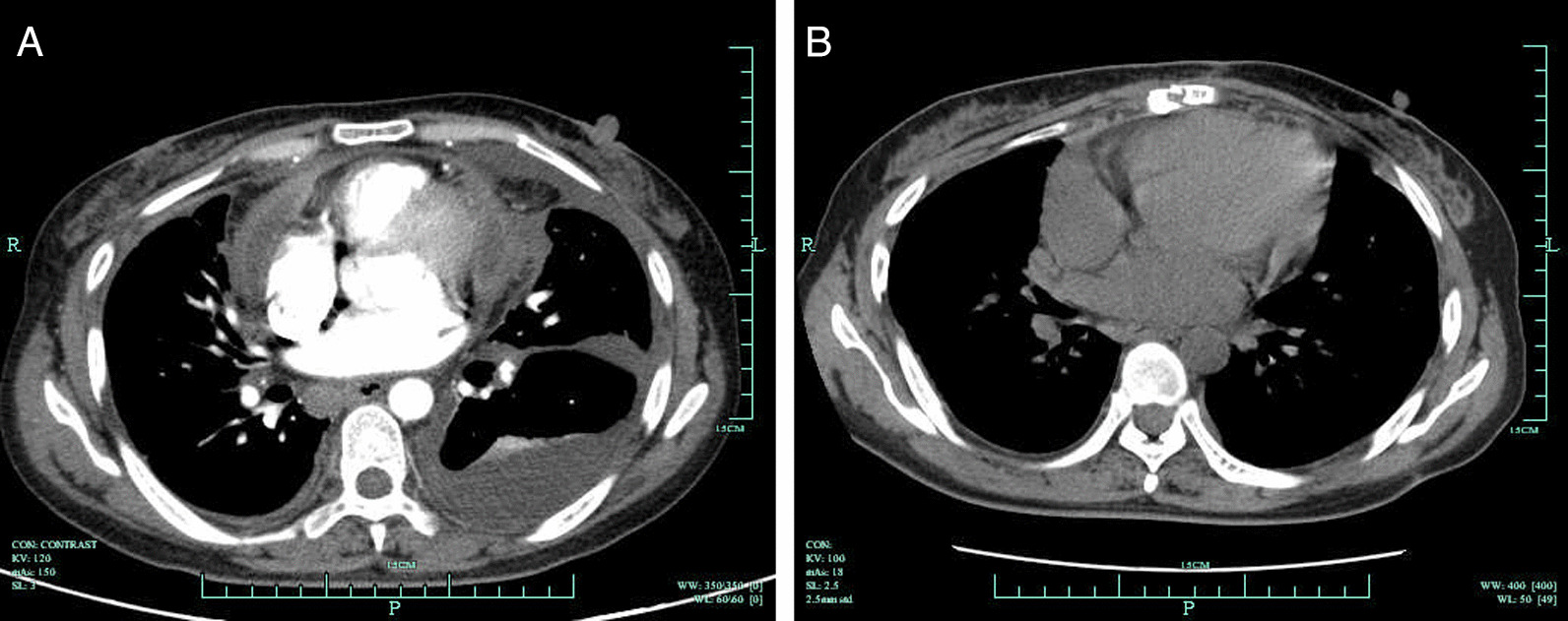
**A** The preoperative CT picture of a patient with constrictive tuberculous pericarditis. **B** The CT picture of the same patient at 2 month postoperatively

Median sternotomy was routinely performed in all cases without the use of cardiopulmonary bypass. The extent of pericardiectomy included at least the anterolateral pericardium between the two phrenic nerves, the basal pericardium over the diaphragmatic surface, the pericardium on the great arteries and the pericardium from superior vena cava-right atrium junction to inferior vena cava-right atrium junction [14]. The preoperative and postoperative CT pictures of a patient in this study were presented in Fig. 1. Postoperative complications were defined as the comorbidities that occurred after surgery but did not exist before.

### Follow-up

The follow-up information was obtained from the hospital outpatient clinic records and the missing records were collected by telephone. Postoperative duration of ATT and the survival condition were mainly recorded during the follow-up period. Overall survival (OS) was defined as the time interval between the date of surgery and the date of death or last follow-up. OS was calculated in months. The last follow-up time was April 2020.

### Statistical analysis

The receiver operating characteristic (ROC) curve of DATT was used to predict postoperative complications and the optimal cutoff value of DATT was determined by calculating the Youden Index. The enrolled patients were divided into two groups according to the cutoff value. The measurement data and numeration data of the two groups were statistically analyzed with t test and χ^2^ test respectively. Kaplan–Meier method and the log-rank test were performed to analyze the survival impact of DATT. All the above analysis was conducted by SPSS software (version 24.0, IBM SPSS Inc. United States). Statistical significance was set at *P* value < 0.05 (All *P* values presented were 2-sided).

## Results

### Optimal cutoff value of DATT

**Fig. 2:**
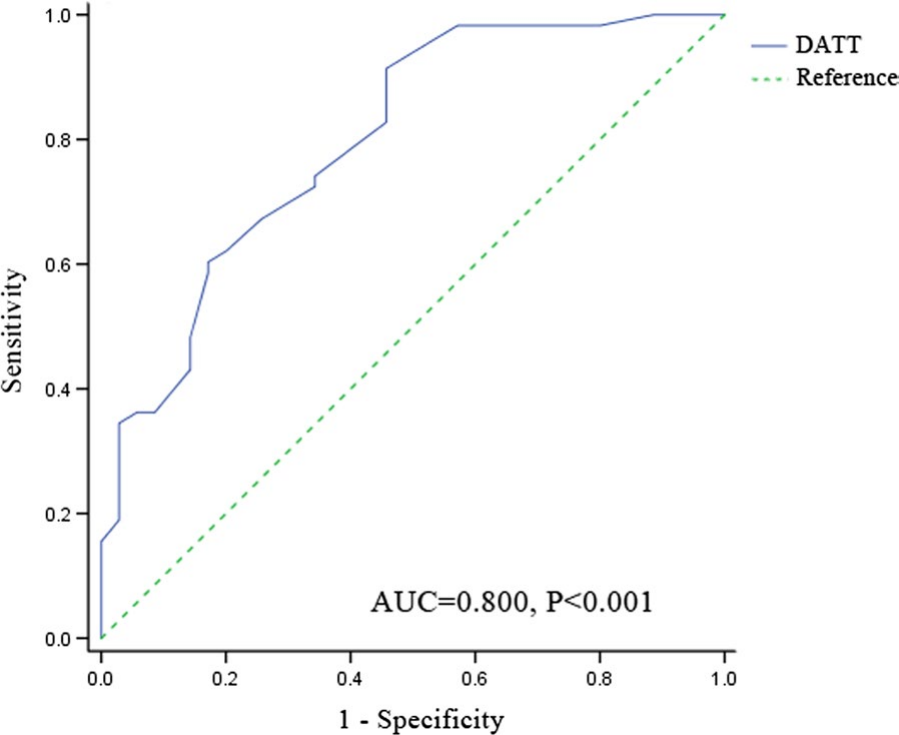
The area under the ROC curve for postoperative complications determined using DATT. *ROC* receiver operating characteristic, *DATT* the duration of anti-tuberculous therapy before pericardiectomy (months), *AUC* area under the curve

The result of ROC curve for predicting postoperative complications showed that the area under curve (AUC) of DATT was 0.800 (95% CI = 0.706–0.893, *P* < 0.001) (Fig. 2). The result showed that there was significant correlation between DATT and postoperative complications. The Youden Index was further calculated and the result showed the optimal cutoff value of DATT was 1.05 months (sensitivity 91.4%, specificity 54.3%, Youden Index 0.457). According to the cutoff value, the patients were divided into the DATT ≤ 1.05 group and the DATT > 1.05 group.

### Baseline characteristics

A total of 93 patients were enrolled in the study, with 24 (25.8%) cases in the DATT ≤ 1.05 group and 69 (74.2%) in the DATT > 1.05 group. The preoperative characteristics of the two groups were presented in the Table 1. There were no statistical differences between the two groups for gender, age, ATT regimens, body mass index (BMI), preoperative central venous pressure (CVP), C-reactive protein (CRP) and other baseline characteristics except erythrocyte sedimentation rate (ESR). The DATT ≤ 1.05 group had higher ESR than the DATT > 1.05 group (*P* = 0.008).

**Table 1:** Characteristics of patients at baseline

Variables	DATT ≤ 1.05 group (N = 24)	DATT > 1.05 group (N = 69)	*P* value
Gender			0.742
Male	18 (75.0%)	54 (78.3%)	
Female	6 (25.0%)	15 (21.7%)	
Age, years	64 (17–83)	58 (16–81)	0.563
ATT regimens			0.198
HRZE	14 (58.3%)	50 (72.5%)	
Other	10 (41.7%)	19 (27.5%)	
Preoperative NYHA functional class			0.396
I	1 (4.2%)	6 (8.7%)	
II	5 (20.8%)	24 (34.8%)	
III	17 (70.8%)	35 (50.7%)	
IV	1 (4.2%)	4 (5.8%)	
Hypertension	2 (8.3%)	12 (17.4%)	0.461
Diabetes	3 (12.5%)	3 (4.3%)	0.359
Atrial fibrillation	4 (16.7%)	11 (15.9%)	1.000
HIV infection	0 (0%)	0 (0%)	/
BMI, kg/m^2^	19.7 (14.9–26.6)	21.1 (16.3–33.1)	0.057
Preoperative CVP, cmH_2_O	30.0 (16.0–42.0)	26.0 (15.5–40.0)	0.278
Pleural effusion	24 (100.0%)	63 (91.3%)	0.312
Ascites	15 (62.5%)	35 (50.7%)	0.319
Pericardial effusion	17 (70.8%)	55 (79.7%)	0.370
Pericardial calcification	5 (20.8%)	17 (24.6%)	0.706
Pericardial thickness, mm	10.9 (5.0–18.9)	10.0 (3.4–18.9)	0.148
LVEF, %	60.9 (51.9–78.0)	57.6 (39.9–74.0)	0.147
Hemoglobin, g/dL	123 (94–151)	122 (90–167)	0.772
CRP, mg/L	19.6 (1.5–99.1)	16.3 (0.8–78.0)	0.206
ESR, mm/h	36.5 (7.0–89.0)	16.5 (2.0–105.0)	0.008
Albumin, g/L	31.7 (24.8–41.7)	33.0 (20.3–48.8)	0.566
Total bilirubin, μmol/L	14.6 (6.4–66.7)	18.8 (3.1–59.2)	0.481
Direct bilirubin, μmol/L	8.8 (3.2–36.4)	11.3 (1.8–50.3)	0.329
Serum creatinine, μmol/L	85.1 (62.8–116.1)	81.2 (44.5–120.6)	0.137
Preoperative BNP, pg/mL	165 (21–961)	169 (21–786)	0.575

Values presented as N (percentage) for categorical variables and median (range) for continuous variables

*DATT* the duration of anti-tuberculous therapy before pericardiectomy (months), *HRZE* isoniazid, rifampicin, pyrazinamide and ethambutol, *NYHA* New York Heart Association, *HIV* human immunodeficiency virus, *BMI* body mass index, *CVP* central venous pressure, *LVEF* left ventricular ejection fraction (measured on echocardiogram), *CRP* C-reactive protein, *ESR* erythrocyte sedimentation rate, *BNP* brain natriuretic peptide

### Short-term outcomes

The comparison of outcomes between the DATT ≤ 1.05 group and the DATT > 1.05 group was shown in the Table 2. The two groups were comparable in the operative duration (*P* = 0.865), the volume of blood loss (*P* = 0.120) and postoperative duration of ATT (*P* = 0.292). Comparing with the DATT ≤ 1.05 group, the incidence of postoperative complications was significantly lower in the DATT > 1.05 group (*P* < 0.001). In addition, the DATT > 1.05 group had shorter postoperative ICU stay (*P* = 0.023), duration of chest drainage (*P* = 0.002) and postoperative hospital stay (*P* = 0.001) in comparison to the DATT ≤ 1.05 group. There was no mortality in the two groups within 30 days after pericardiectomy.

**Table 2:** Comparison of outcomes between DATT ≤ 1 and DATT > 1 groups

Variables	DATT ≤ 1.05 group (N = 24)	DATT > 1.05 group (N = 69)	*P* value
Operative duration, min	243 (157–375)	245 (115–400)	0.865
Blood loss, mL	100 (40–300)	200 (50–800)	0.120
Postoperative complications	19 (79.2%)	16 (23.2%)	< 0.001
Postoperative ICU stay, days	3.5 (0–8)	2 (0–11)	0.023
Duration of chest drainage, days	16.5 (5–32)	10 (4–52)	0.002
Postoperative hospital stay, days	22 (7–48)	15 (9–60)	0.001
Mortality within 30 days	0 (0.0%)	0 (0.0%)	/
Postoperative duration of ATT, months	10 (3–18)	12 (0–30)	0.292

Values presented as median (range) for continuous variables and N (percentage) for categorical variables

*DATT* the duration of anti-tuberculous therapy before pericardiectomy (months), *ICU* intensive care unit, *ATT* anti-tuberculous therapy

### Survival impact

**Fig. 3:**
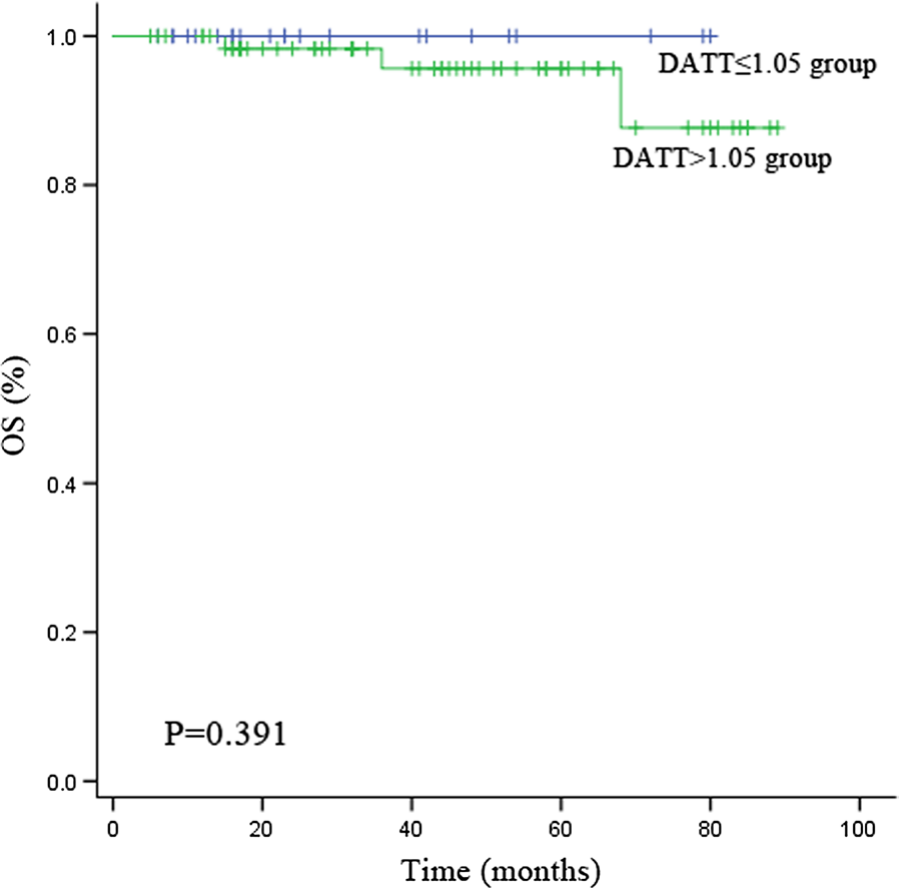
Kaplan–Meier curves comparing OS between the DATT ≤ 1.05 group and the DATT > 1.05 group. *DATT* the duration of anti-tuberculous therapy before pericardiectomy (months)

Follow-up information was successfully collected in 88 (94.6%) patients and 5 (5.4%) patients were lost to contact after surgery. The median follow-up time was 38 months, ranging from 5 to 89 months. There was no recurrence of constriction from residual pericardium during the follow-up period. The Kaplan–Meier curve and the log-rank test showed that there was no difference in the OS between the DATT ≤ 1.05 group and the DATT > 1.05 group (*P* = 0.391) (Fig. 3).

## Discussion

Although the overall incidence of constrictive pericarditis has not been investigated, it appears to be relatively rare worldwide [9]. Tuberculosis has remained the dominant cause of constrictive pericarditis in the developing areas with the rate ranging from 23 to 91% [15, 16]. Constrictive tuberculous pericarditis should deserve more attention due to its unfavorable outcomes. The current standard anti-tuberculous regimen of first-line drugs (isoniazid, rifampin, pyrazinamide, and ethambutol) achieves satisfactory cure rate in pulmonary tuberculosis [17], but its effect on the constrictive tuberculous pericarditis has been unclear. There have been several studies indicating that the concentration of anti-tuberculous drugs is low in the pericardial space because of poor penetration, especially rifampin and pyrazinamide [18–20]. Due to the inadequate concentration of primary sterilizing effect drugs, the effect of anti-tuberculous treatment on the development of constrictive pericarditis is limited [8], which suggests that constrictive tuberculous pericarditis is chronic and progressive in most cases. As a result, medication therapy is only a palliative and temporary approach while surgical pericardiectomy is the definitive treatment to relive the constriction and improve clinical symptoms [21, 22].

However, pericardiectomy is associated with high morbidity and mortality [12]. In order to reduce the incidence of postoperative complication and death, some studies have attempted to find the potential risk factors to improve the strategies of perioperative management. The extent of pericardial resection was one of the risk factors and complete pericardiectomy was associated with superior surgical outcomes to partial pericardiectomy [16, 23, 24]. Cardiopulmonary bypass was also a predictor of poor prognosis and the 30-day mortality rate might reduce without the use of cardiopulmonary bypass [25]. In our study, all patients were performed complete pericardiectomy without the use of cardiopulmonary bypass and there was no in-hospital death. In addition to comorbidities, organ functional conditions and etiology of constriction, surgical outcomes were also affected by the timing of pericardiectomy [26, 27]. Early surgical intervention was crucial to reduce mortality and morbidity [26, 28]. In traditional practice, pericardiectomy was often performed after ATT in constrictive tuberculous pericarditis [8], but the appropriate DATT was unclear.

This study first explored the optimal time interval between the initiation of ATT and pericardiectomy in the patients with constrictive tuberculous pericarditis. We found that 1.05 (months) was the optimal cutoff value of DATT. Shorter postoperative ICU stay and hospital stay were observed in the DATT > 1.05 group, as well as the duration of chest drainage. In contrast, there was no statistical difference between the two groups in the 30-day mortality after surgery, recurrence and long-term survival. These results indicated that it would be of potential benefit to enhance recovery after surgery if DATT lasted for at least 1 month.

Our study has several limitations. First, this is a single-center retrospective study, so the selection bias is inevitable. Second, due to the relatively small sample size, we only find the lower boundary of DATT but fail to find the upper boundary which requires further studies. Finally, some valuable characteristics such as the extent of pericardial adhesion are failed to be recorded due to the retrospective design.

## Conclusion

It would be of potential benefit to enhance recovery after pericardiectomy in the patients with constrictive tuberculous pericarditis if the duration of anti-tuberculous therapy before surgery lasted for at least 1 month without increasing postoperative recurrence and mortality.

## Data Availability

The datasets used and/or analyzed during the current study are available from the corresponding author on reasonable request.

## Abbreviations

DATT: The duration of anti-tuberculous therapy before pericardiectomy
ATT: Anti-tuberculous therapy
HRZE: Isoniazid, rifampicin, pyrazinamide and ethambutol
OS: Overall survival
ROC: Receiver operating characteristic
AUC: Area under curve
NYHA: New York Heart Association
HIV: Human immunodeficiency virus
BMI: Body mass index
CVP: Central venous pressure
LVEF: Left ventricular ejection fraction
CRP: C-reactive protein
ESR: Erythrocyte sedimentation rate
BNP: Brain natriuretic peptide
ICU: Intensive care unit
